# Parenting Style, the Home Environment, and Screen Time of 5-Year-Old Children; The ‘Be Active, Eat Right’ Study

**DOI:** 10.1371/journal.pone.0088486

**Published:** 2014-02-12

**Authors:** Lydian Veldhuis, Amy van Grieken, Carry M. Renders, Remy A. HiraSing, Hein Raat

**Affiliations:** 1 Department of Public and Occupational Health, EMGO Institute for Health and Care Research, VU University Medical Centre, Amsterdam, The Netherlands; 2 Department of Public Health, Erasmus MC – University Medical Center Rotterdam, Rotterdam, The Netherlands; 3 Institute of Health Sciences, Faculty of Earth and Life Sciences, VU University Amsterdam, Amsterdam, The Netherlands; University of Akron, United States of America

## Abstract

**Introduction:**

The global increase in childhood overweight and obesity has been ascribed partly to increases in children's screen time. Parents have a large influence on their children's screen time. Studies investigating parenting and early childhood screen time are limited. In this study, we investigated associations of parenting style and the social and physical home environment on watching TV and using computers or game consoles among 5-year-old children.

**Methods:**

This study uses baseline data concerning 5-year-old children (n = 3067) collected for the ‘Be active, eat right’ study.

**Results:**

Children of parents with a higher score on the parenting style dimension involvement, were more likely to spend >30 min/day on computers or game consoles. Overall, families with an authoritative or authoritarian parenting style had lower percentages of children's screen time compared to families with an indulgent or neglectful style, but no significant difference in OR was found. In families with rules about screen time, children were less likely to watch TV>2 hrs/day and more likely to spend >30 min/day on computers or game consoles. The number of TVs and computers or game consoles in the household was positively associated with screen time, and children with a TV or computer or game console in their bedroom were more likely to watch TV>2 hrs/day or spend >30 min/day on computers or game consoles.

**Conclusion:**

The magnitude of the association between parenting style and screen time of 5-year-olds was found to be relatively modest. The associations found between the social and physical environment and children's screen time are independent of parenting style. Interventions to reduce children's screen time might be most effective when they support parents specifically with introducing family rules related to screen time and prevent the presence of a TV or computer or game console in the child's room.

## Introduction

The global increase in prevalence of childhood overweight and obesity has been ascribed to several trends including the increase in consumption of energy-dense diets and the increase in sedentary behavior (in particular the increase in screen time; time spent watching TV and on computers or game consoles) [1], [2], [3]. Children's screen time increases with age and patterns of screen time appear to be stable over time [4], [5]. Parents influence their children's screen time by their practices (e.g. having rules about watching TV) and by controlling the physical home environment (e.g. placing or not allowing a TV in the child's bedroom) [1], [6].

Interventions aiming to reduce children's screen time should be family-based, start during early childhood, and target modifiable factors in the home setting [1], [7]. It is likely that the home environment factors that influence children's screen time, and their impact on screen time, change during childhood [4], [8]. Most studies investigating associations between the social and physical home environment and children's screen time included school-aged children (between the age of 6 to 13 years) [4], [9], [10], [11], [12], [13], [14], [15], [16], [17], [18]; studies investigating these associations in children below 6 years of age are limited [19], [20], [21]. Previous studies found that family rules on watching TV are associated with less TV viewing [10], [13], [14], [20] and that high child autonomy is associated with more TV viewing [9], [15]. The results of studies investigating the influence of having a TV in the child's bedroom on the amount of TV viewing are inconsistent; some studies found that a TV in the child's bedroom was associated with increased TV viewing [12], [14], [16], [19], whereas others found no association [4], [10], [11], [15]. Further, most studies included only watching TV as a screen time activity and only a few studies included using computers or game consoles as screen time [15], [18], [20], [22].

Parenting practices and parenting decisions on the physical home environment take place in the context of the parenting style (i.e. the climate in which a family functions and children are raised) [8], [23]. Parenting style can be categorized as authoritative, authoritarian, indulgent, or neglectful [24]. However, the relationships between parenting style, the social and physical home environment and children's screen time remain largely unknown [25], [26], [27].

**Figure 1:**
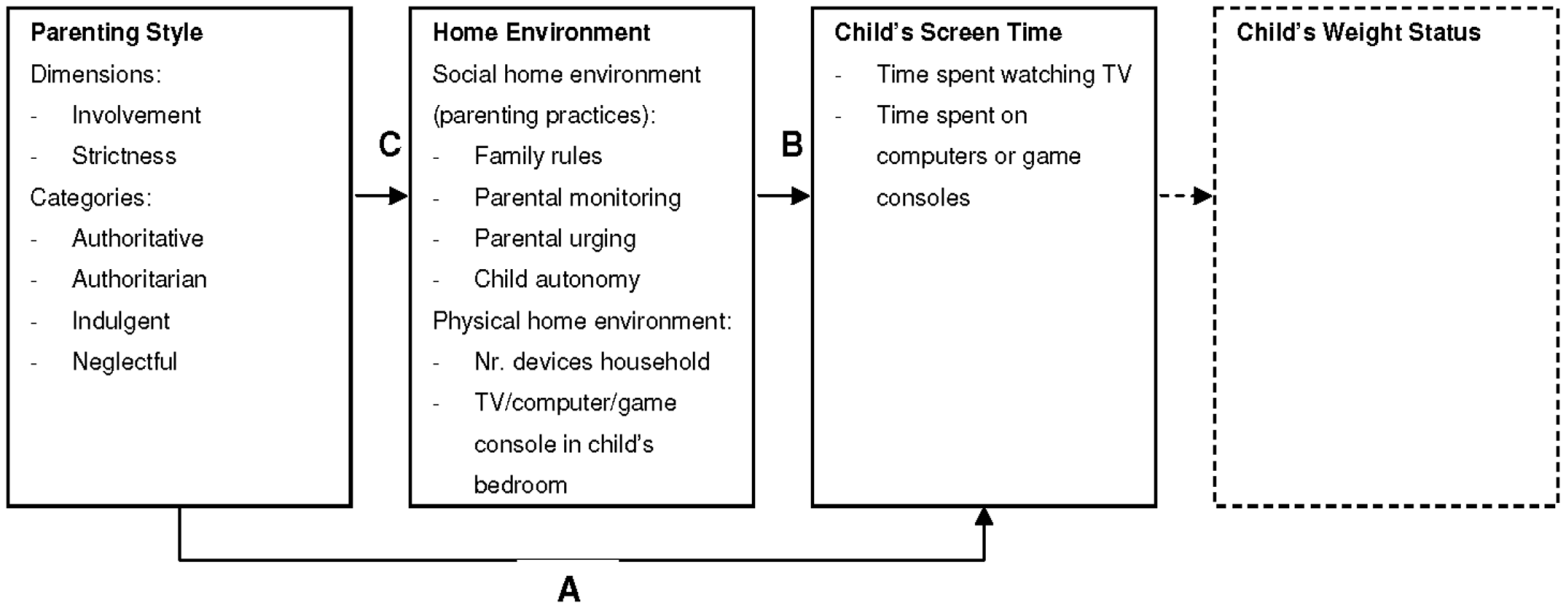
Hypothesized model of relationships between parenting style, home environment, children's screen time and weight status.

The relationships between parenting style, the home environment, and children's screen time and weight status are complex. It is unclear how parenting style and the home environment are associated with young children's screen time. In this study, we investigated associations between parenting style, the home environment and screen time among a large sample of 5-year-old children (Figure 1; the association with children's weight status is outside of the scope of the present study). First, we hypothesized that screen time would be lower for children of parents with higher scores on strictness in general (parents with an authoritarian or authoritative parenting style) (arrow A in Figure 1). Second, we hypothesized that children's screen time would be the lowest for children in families with rules regarding screen time and would be the highest for children with a TV or computer or game console in their bedroom (arrow B in Figure 1). Thirdly, we also examined whether the association between parenting style and children's screen time was mediated through the home environment (arrow C and B in Figure 1).

## Methods

### Design and study population

This study is embedded in the ‘Be active, eat right’ study. As detailed elsewhere [28], the ‘Be active, eat right’ study aims to assess the effects of an overweight prevention program among children at elementary schools throughout the Netherlands. The Medical Ethics Committee of the Erasmus MC - University Medical Centre Rotterdam approved the study protocol. Of the 37 municipal health services in the Netherlands, nine municipal health services agreed to participate in the study. A total of 13,638 parents of 5-year-olds were invited by mail for a free-of charge well-child visit at one of these nine municipal health services and 64.4% (n = 8784) provided written informed consent to participate in the study. The children and their parents were randomly allocated into either an intervention group or a control group. Baseline data were collected during the 2007–2008 school year and these data were used for the present study.

Parents completed questionnaires with items on socio-demographic characteristics and lifestyle-related characteristics pertaining to themselves and their child. To minimize the respondent burden, only a subgroup (n = 4381) of the total population (n = 8784) included in the study was asked to complete an additional questionnaire. This additional questionnaire included items on parenting style, and the social and physical home environment. All parents in the control group were asked to complete this questionnaire (n = 3942) whereas only parents of children with overweight or obesity in the intervention group were asked to complete the questionnaire (n = 439) [28]. The questionnaire was developed based on items used in other studies on screen time, parenting style, and social and physical home environment characteristics [29], [30], [31]. The response rate for the questionnaire was 74.8% (n = 3278). After removing records with missing data on the child's screen time (n = 211), a study population of n = 3067 children and their parents remained.

### Screen time of the children

Parents reported on a questionnaire the time their child spent watching TV and using a computer or game console. We indicated in the questionnaire that a computer or game console also included portable consoles. Parents were asked to indicate the average number of weekdays and weekend days their child spent time on a computer or game console and watching TV, and how much time their child spent on a computer or game console and watched TV on average in the morning, the afternoon, and at night after dinner on weekdays and during weekends. We combined the weekday and weekend data and recoded the two screen time variables. To dichotomize using computers or game consoles, we used 30 minutes per day (min/day) as the cut-off point to allow meaningful comparisons between subgroups that spent ≤30 min/day versus >30 min/day on computers or game consoles (approximately 15% of the children spent >30 min/day on computers or game consoles; <5% spent >1 hour/day on computers or game consoles). Watching TV was dichotomized based on international recommendations [32], [33], [34] into watching TV≤2 hours per day (hrs/day) or >2 hrs/day.

### Parenting style

Parenting style was assessed using an adapted version of the Steinberg instrument, which is considered one of the best measurement tools available to measure parenting style [8], . Two parenting style dimensions were measured: involvement and strictness of the parents in general. The involvement and strictness scales included nine and six items, respectively. Parents responded on a 5-point scale with the scale ranging from strongly agree to strongly disagree. Internal consistencies were α = 0.75 for the involvement scale and α = 0.78 for the strictness scale. A full description of scales, scale properties, items, and item response scales is available in Appendix S1.

In the main analyses, we used the continuous parenting style dimensions involvement and strictness [35]. The involvement and strictness scales can be used to define four parenting styles: authoritative (high on involvement and high on strictness), authoritarian (low on involvement and high on strictness), indulgent (high on involvement and low on strictness) and neglectful (low on involvement and low on strictness). For interpretation purposes we categorized parents into the four styles by using the median splits on both the involvement and strictness scales [24].

### Social environment and physical home environment

The following parenting practices (i.e. the social environment) regarding screen time were measured: family rules regarding screen time, parental monitoring of their child's screen time, whether the parents urge their child to turn off the TV or computer or game console, and the autonomy of the child regarding screen time. A ‘rules’ index was created by summing the number of rules, with a higher score indicating that the parents had more rules with regard to their child's screen time. Parental monitoring and urging to turn off the TV or computer or game console were assessed using a 5-point response scale. A higher score on each of these items indicated that the parents monitor their child's screen time and urge their child to turn off the TV or computer or game console. Child autonomy was assessed using three items. A scale was created, with higher scores indicating more autonomy of the child concerning screen time.

The physical home environment was measured using two items; the number of TVs and computers or game consoles present in the household, and whether the child has a TV or computer or game console in his or her bedroom.

A full description of scales, scale properties, items, and item response scales is available in Appendix S1. We looked for presence of collinearity between the variables in the groups measuring the social environmental and physical environment [36]. For all variables the value of the variance inflation factor (VIF) [37] was close to 1 (range 1.07–1.21), indicating that none of the variables had strong linear relationships with the others variables. Based on these results, we concluded that there were no indications for multicollinearity.

### Sociodemographic characteristics

We included several potential confounding sociodemographic characteristics in this study: child sex and the child's ethnic background (Dutch, non-Dutch), parental educational level (high, mid, or low), family structure (two-parent family, single-parent family or other), and parental employment status (employed full-time/part-time or not employed). A child was considered to be of non-Dutch ethnic background when at least one of the parents was born abroad (definition as used by Statistics Netherlands) [38]. Parental education level was recoded in three categories according to the Dutch standard classification as defined by Statistics Netherlands [39]: high level (academic higher education/university education, higher professional education), mid level (pre-university education, senior secondary education, and senior secondary vocational education), and low level (preparatory secondary vocational education, lower secondary vocational education, primary education, and no education).

### Statistical analysis

Mean and frequency differences in sociodemographic characteristics between the subgroups of parent-reported screen time were examined using t-tests for continuous variables and Chi-square statistics for categorical variables. We examined differences in children's screen time by parenting styles using Chi-square statistics. We used multivariable logistic regression analyses to test the associations between parenting style, the home environment and children's screen time. We report the odds ratios (ORs) and 95% confidence intervals (CIs) for all models.

First, we tested the association between the parenting style dimensions, parenting style categories, and the child's screen time. Second, we tested the associations between the social and physical home environment characteristics and the child's screen time. Further, to test whether the association between parenting style and the child's screen time (basic model) was mediated by the home environment, we adjusted the basic model for each social and physical home environment characteristic one at a time.

Additionally, we also checked for potential effect-modification by the physical home environment characteristics or the sociodemographic characteristics in the associations between practices (i.e. the social environment) and the child's screen time. No significant interactions were found for the physical environment characteristics and no consistent interactions were found for the sociodemographic characteristics. We therefore decided not to stratify the analyses. We adjusted the analyses for the sociodemographic characteristics (sex and age of the child, child's ethnic background, educational level of the parent, parent employment status, and family structure).

Only children with complete data concerning screen time were included for analyses. Of all other variables included in the study, the percentages of missing values ranged from 0.1–11.9 with approximately two-thirds of the variables having <5% missing values. Because the missing values were not completely at random, we used the multiple imputation procedure in SPSS (version 20.0). The imputation procedure was carried out using all variables in the study except parent age and sex of the parent. All analyses were performed on both the original dataset with complete cases [40] and the five imputed datasets and were then compared. Because there were no differences in the direction of the associations found, the ORs and their CIs presented are the pooled results of the analyses performed on the imputed datasets.

We performed the analyses using SPSS 20 for Windows (International Business Machines (IBM) Corp., SPSS Statistics, version 20.0, Armonk, New York, USA).

## Results

Table 1 shows the general characteristics of the parents and children included in the study. Mean age of the children in the study population (total n = 3067) was 5.8 (SD 0.4) years and 49.3% were male. Children with a mother with a low educational level and children of non-Dutch ethnic background were more likely to watch TV>2 hrs/day and spend >30 min/day on computers or game consoles. Further, children in single-parent families were more likely to watch TV>2 hrs/day and boys were more likely to spend >30 min/day on computers or game consoles.

**Table 1:** Characteristics of the total study population (n = 3067) and by amount of time watching TV (TV≤2 hrs/day versus >2 hrs/day) and by amount of time using computers or game consoles (≤30 min/day versus >30 min/day).

	Total	Watching TV			Using computers or game consoles		
		≤2 hrs/day (n = 2419)	>2 hrs/day (n = 648)	*p*-value^a^	≤30 min/day (n = 2601)	>30 min/day (n = 466)	*p*-value^a^
**Parent characteristics**							
Mean age, years (SD)	36.9 (4.6)	37.0 (4.4)	36.6 (5.1)	0.12	36.8 (4.5)	37.1 (5.0)	0.21
Mother is respondent, n (%)	2762 (90.5)	2185 (90.8)	577 (89.5)	0.31	2347 (90.7)	415 (89.6)	0.50
Low educational level^b^, n (%)	579 (19.4)	370 (15.7)	209 (33.0)	<0.001	434 (17.2)	145 (32.0)	<0.001
Not employed, n (%)	929 (33.1)	732 (33.2)	197 (32.8)	0.98	778 (32.8)	151 (35.0)	0.45
Single parent, n (%)	183 (6.0)	120 (5.0)	63 (9.8)	<0.001	151 (5.8)	32 (6.9)	0.41
**Child characteristics**							
Mean age, years (SD)	5.8 (0.4)	5.8 (0.4)	5.8 (0.4)	0.11	5.8 (0.4)	5.9 (0.4)	<0.001
Boy, n (%)	1509 (49.3)	1187 (49.2)	322 (49.7)	0.82	1205 (46.4)	304 (65.2)	<0.001
Non-Dutch ethnic background, n (%)	321 (11.1)	210 (9.1)	111 (18.8)	<0.001	248 (10.1)	73 (16.7)	<0.001
**Child screen time**							
Watching TV, min/day (SD)	86.3 (53.2)	65.2 (31.8)	165.0 (42.2)	<0.001	80.1 (49.1)	120.9 (61.4)	<0.001
Using computers or game consoles, min/day (SD)	17.0 (23.2)	13.8 (17.2)	29.3 (35.4)	<0.001	9.5 (8.5)	59.3 (32.0)	<0.001

The means and frequencies presented are means and frequencies of the original dataset. Missing values were 16 (0.5%) for age of the parent, 15 (0.5%) for sex of the parent, 84 (2.7%) for educational level, 17 (0.6%) for family structure, 264 (11.9%) for employment status of the parent, 239 (7.8%) for age of the child, 4 (0.1%) for sex of the child, and 182 (5.9%) for ethnic background of the child.

a T-tests were used for continuous variables and Chi-square statistics were used for categorical variables to examine differences between the subgroups of children that watch TV≤2 hrs/day versus watch TV>2 hrs/day and between the subgroups of children that use computers or game consoles ≤30 min/day versus >30 min/day on computers or game consoles; the *p*-values are the pooled results of analysis of the five imputed datasets.

b Low education = no education, primary education, lower secondary vocational education, and preparatory secondary vocational education; mid education = senior secondary vocational education, senior secondary education, and pre-university education; high education = higher professional education, academic higher education (university education).

In Table 2, the associations between parenting style and parent-reported screen time of the children are presented. Children of parents with a higher score on the parenting style dimension involvement were more likely to spend >30 min/day on computers or game consoles (1.34 (95% CI: 1.02–1.77)). Overall, families with an authoritative or authoritarian parenting style had lower percentages of children's screen time compared to families with an indulgent or neglectful parenting style. However, no differences in ORs were found between subgroups with an authoritative parenting style and subgroups with another parenting style.

**Table 2:** Logistic regression analyses for association between parenting style and children's screen time (n = 3067).

	Watching TV>2 hrs/day		Using computers or game consoles >30 min/day	
		OR (95% CI)		OR (95% CI)
**Parenting style dimensions**	**Mean (SD)**		**Mean (SD)**	
Involvement	4.4 (0.4)	0.88 (0.70–1.12)	4.4 (0.4)	**1.34 (1.02–1.77)**
Strictness	4.4 (0.6)	0.92 (0.79–1.07)	4.4 (0.6)	1.10 (0.92–1.31)
**Parenting style categories**	**n (%)** *		**n (%)** **	
Authoritative (n = 1061)	202 (19.0)	1.00 (ref)	166 (15.6)	1.00 (ref)
Authoritarian (n = 399)	74 (18.5)	1.05 (0.78–1.43)	43 (10.8)	0.70 (0.48–1.03)
Indulgent (n = 426)	106 (24.9)	1.22 (0.92–1.62)	80 (18.8)	1.09 (0.79–1.51)
Neglectful (n = 929)	223 (24.0)	1.20 (0.96–1.49)	144 (15.5)	0.87 (0.68–1.12)

For details on the measures used, see Appendix S1.

The means and frequencies presented are means and frequencies of the original dataset. Missing values were 252 (8.2%) for parenting style. To examine differences in watching TV and using computers or game consoles across parenting styles, Chi-square statistics were used; the p-values are the pooled results of analysis of the five imputed datasets.

The ORs are adjusted for sociodemographic characteristics (sex and age of the child, ethnic background of the child, educational level of the parent, employment status and family structure).

* *p*<0.05 for difference across parenting styles.

** *p*<0.01 for difference across parenting styles.

In Table 3 and Table 4, the associations between the characteristics of the social and physical home environment and children's screen time are presented. For example, children in families with rules about when and how long children are allowed to watch TV (present in 69.1% of all families) had an OR of 0.60 (95% CI: 0.47–0.76) for watching TV>2 hrs/day compared to children without these family rules. Children in families with rules about when and how long children are allowed to use computers or game consoles (present in 61.8% of the families) had an OR of 1.91 (95% CI: 1.47–2.48) for spending >30 min/day on computers or game consoles. For children with higher autonomy regarding using computers or game consoles, the OR was 1.50 (95% CI: 1.36–1.66). Further, the number of TVs and computers or game consoles present in the household was positively associated with children's screen time, and children who have a TV or computer or game console in their bedroom had higher odds for watching TV>2 hrs/day and spending >30 min/day on computers or game consoles.

**Table 3:** Logistic regression analyses for associations between home environment characteristics and watching TV by the child (n = 3067).

		Watching TV>2 hs/day, OR (95% CI)
**Social home environment (parenting practices)**		
Nr. of family rules about watching TV^a^, n (%)		
1 rule (when or how long the child is allowed to watch TV)	457 (15.1)	0.91 (0.67–1.22)
2 rules (when and how long the child is allowed to watch TV)	2084 (69.1)	**0.60 (0.47–0.76)**
Parental monitoring concerning watching TV, always/often^b^, n (%)	2596 (86.0)	**0.55 (0.43–0.69)**
Parental urging to turn off TV, always/often^b^, n (%)	1267 (42.1)	0.94 (0.77–1.13)
Child autonomy concerning watching TV^c^, mean (SD)	2.2 (0.9)	**1.55 (1.40–1.70)**
**Physical home environment**		
Nr. of TVs in household^d^, n (%)		
1 TV	913 (31.3)	**1.00**
2–3 TVs	1872 (61.2)	**1.79 (1.44–2.23)**
≥4 TVs	128 (4.2)	**2.83 (1.85–4.32)**
Child has TV in bedroom, yes^e^, n (%)	266 (8.7)	**2.62 (2.00–3.44)**

For details on the measures used, see Appendix S1.

The frequencies (n (%)) and means presented are frequencies and means of the original dataset. Missing values were 49 (1.6%) for family rules about watching TV, 47 (1.5%) for parental monitoring, 55 (1.8%) for parental urging to turn off the TV, 73 (2.4%) for child autonomy concerning watching TV, 7 (0.2%) for number of TVs in the household, and 12 (0.4%) for whether the child has a TV in the bedroom.

The ORs are the pooled results of analysis of the five imputed datasets.

The ORs are adjusted for sociodemographic characteristics (sex and age of the child, ethnic background of the child, educational level of the parent, employment status and family structure).

a The reference category (OR = 1.00) is ‘no rules’.

b The reference category (OR = 1.00) is ‘never, seldom, or sometimes’.

c An increase on child autonomy indicates higher autonomy of the child concerning screen time.

d Households without a TV (n = 147, 4.8%) were excluded from analysis.

e The reference category (OR = 1.00) is ‘no’.

**Table 4:** Logistic regression analyses for associations between home environment characteristics and using computers or game consoles by the child (n = 3067.

		Using computers or game consoles >30 min/day, OR (95% CI)
**Social home environment (parenting practices)**		
Nr. of family rules about using computers or game consoles^a^, n (%)		
1 rule (when or how long the child is allowed to use a computer or game console)	232 (7.9)	**1.80 (1.17–2.77)**
2 rules (when and how long the child is allowed to use a computer or game console)	1823 (61.8)	**1.91 (1.47–2.48)**
Parental monitoring concerning using computers or game consoles, always/often^b^, n (%)	2353 (80.5)	**1.60 (1.20–2.12)**
Parental urging to turn off computer or game console, always/often^b^, n (%)	876 (30.0)	**2.34 (1.89–2.90)**
Child autonomy concerning using computers or game consoles^c^, mean (SD)	2.0 (1.7)	**1.50 (1.36–1.66)**
**Physical home environment**		
Nr. of computers or game consoles in household^d^, n (%)		
1 computer	1208 (39.6)	**1.00**
2–3 computers	1191 (39.1)	**1.91 (1.51–2.42)**
≥4 computers	259 (8.5)	**3.64 (2.62–5.07)**
Child has computer or game console in bedroom, yes^e^, n (%)	468 (15.3)	**2.57 (2.03–3.25)**

For details on the measures used, see Appendix S1.

The frequencies (n (%)) and means presented are frequencies and means of the original dataset. Missing values were 116 (3.8%) for family rules about using computers or game consoles, 145 (4.7%) for parental monitoring, 146 (4.8%) for parental urging to turn off the computer or game console, 158 (5.2%) for child autonomy concerning using computers or game consoles, 19 (0.6%) for number of computers or game consoles in the household, and 10 (0.3%) for whether the child has a computer or game console in the bedroom.

The ORs are the pooled results of analysis of the five imputed datasets.

The ORs are adjusted for sociodemographic characteristics (sex and age of the child, ethnic background of the child, educational level of the parent, employment status and family structure).

a The reference category (OR = 1.00) is ‘no rules’.

b The reference category (OR = 1.00) is ‘never, seldom, or sometimes’.

c An increase on child autonomy indicates higher autonomy of the child concerning screen time.

d Households without a computer or game console (n = 390, 2.7%) were excluded from analysis.

e The reference category (OR = 1.00) is ‘no’.

We found a statistically significant association between the parenting style dimension involvement and using computers or game consoles by the child (Table 2), and tested whether this association was mediated by the home environment. Adding the home environment characteristics to the model changed the OR in the range between 2.9% and 23.5% (Appendix S2). After adjustment for the relevant home environment characteristics (characteristics that changed the OR >10%), the association between the parenting style dimension involvement and use of computers or game consoles by the child was no longer statistically significant (OR 1.30 (95% CI: 0.98–1.72), data not shown).

## Discussion

In this study among more than three thousand 5-year-old children from different parts of the Netherlands, we investigated associations between parenting style, the home environment, and parent-reported screen time. First, as hypothesized, children's screen time was lower for children in families with an authoritative or authoritarian parenting style compared to children in families with an indulgent or neglectful parenting style. However, we only found a statistically significant association between the parenting style dimension involvement and using computers or game consoles by the child (children with parents with higher involvement, were more likely to spend >30 min/day on computers or game consoles). No differences in ORs were found between subgroups with an authoritative parenting style and subgroups with another parenting style. Second, as hypothesized, we found that children in families with rules and parental monitoring regarding watching TV are less likely to watch TV>2 hrs/day and that children with higher autonomy regarding watching TV are more likely to watch TV>2 hrs/day. Further, having multiple TVs within the household and a TV in the child's bedroom is associated with higher odds for watching TV>2 hrs/day. Overall, the results for spending >30 min/day on computers or game consoles were comparable to these results for watching TV>2 hrs/day. Thirdly, we found that characteristics of the social home environment mediated the association between the parenting style dimension involvement and children's use of computers or game consoles.

We found that children are more likely to spend >30 min/day on computers or game consoles in families where rules are present concerning using computers or game consoles, where parents urge the child to turn off the computer or game console, and where parents monitor the time a child uses computers or game consoles. The directions of these associations are unlike those for watching TV and were not as expected. However, as we used cross-sectional data, the direction of these associations might be the other way around. In other words, it might be that parents have rules about the amount of computer use and monitor the time their child uses computers or game consoles *because* the child was spending relatively large amounts of time on computers or game consoles. From our data, 1920 (62.6%) children in the study population spent less than 15 minutes a day on a computer or game console, and 617 of these children (equal to 20.1% of the total study population) did not spend any time on a computer or game console. We repeated the analyses after excluding the children who spent no time on a computer or game console. The higher odds for spending >30 min/day on computers or game consoles was no longer statistically significant for children with parental monitoring and with 1 family rule about using computers or game consoles.

Our new hypothesis that a child spending a relatively high amount of time on computers or game consoles leads to family rules about amount of computer use is strengthened by the finding that children with high autonomy regarding using computers or game consoles are also more likely to spend >30 min/day on computers or game consoles. However, over the past few years there has been an increase in the use of electronic media by very young children [41] and with the introduction of smart phones and tablets more parents are probably introducing rules on the amount of time a child may spend using a device.

In the main analyses, we chose to use the continuous parenting style dimensions of strictness and involvement instead of a categorization in the four parenting styles, as this categorization is arbitrary, sample-specific, and causes reduction in measurement reliability [35], [42], [43]. We also investigated the effect of the two parenting style dimensions in combination, and the interactions appeared to be non-significant (p-values>0.1, data not shown). In our study, we only used the categorization into the four parenting styles for interpretation purposes. To categorize parents, we dichotomized the strictness and the involvement scales based on the median values of both scales in our study population [24]. Other studies defined the four parenting categories also by ‘trichotomizing’ both scales using tertiles (which presumably leads to more distinct parenting style groups compared to using dichotomization, as parents who score in the middle tertile are excluded from the analyses), or by using cut-off points for the scales [44]. For comparison; by using trichotomisation in our study population, 16.3% of the parents were classified authoritative, 1.3% as authoritarian, 4.4% as indulgent, and 18.5% as neglectful. By using the cut-off points of Steinberg et al, 9.4% of the parents were classified authoritative, 15.6% as authoritarian, 5.9% as indulgent, and 69.1% as neglectful. We recommend future studies to investigate cluster analytic approaches when categorizing parents into parenting styles [45].

Other methodological considerations of the present study need to be addressed also. As we used cross-sectional data, the direction of the associations can not be confirmed. Further, child behavior was based on data reported by the parent. Parents might have given socially desirable answers even though anonymity was assured. Parent-reports are also susceptible to recall bias. However, by asking parents about their child's screen time on week days and weekend days separately, we took into account potential variation in screen time between weekdays and weekend days. Parents were asked to report the time their child spent watching TV and using computers or game consoles during an average week in total; we did not differentiate between households in the questionnaire. We did, however, adjust the analyses for family structure (two-parent family, single-parent family or other). To minimize the respondent burden, only one questionnaire was obtained per child, and in most cases this questionnaire was completed by the child's mother (90.5%). It was not possible in the present study to compare, for example, parenting style of the mother and the father. Further, the prevalence of overweight and obesity was relatively high in our study population, because all parents in the control group were asked to complete the questionnaire whereas only the parents of children with overweight or obesity in the intervention group were asked to complete the questionnaire [28]. The results reported in this study were the same when we repeated the main analyses and included the control group only. Based on this, we conclude that the relatively high prevalence of overweight and obesity in our study population did not affect the results reported in this study.

Our results support the evidence emerging from the literature of modifiable factors in the home environment that are associated with the time children spend watching TV or using computers or game consoles. The strengths of our study are that we included a large study population of young children with a small age range, therefore our results are specific to the 5-year-old age group. Further, we included two indicators of screen time (watching TV and using computers or game consoles) and analyzed the data separately. It has been recommended that watching TV and using computers or game consoles should be investigated separately and not be combined as one screen-time variable as these behaviors relate differently to energy intake and energy expenditure [22]. The opposite associations we found between family rules and watching TV and family rules and using computers or game consoles further supports the need to investigate these indicators of screen time separately.

To our knowledge, our study is the first to investigate associations between parenting style, the home environment, and children's screen time. Although children in families with an authoritative and authoritarian parenting style had the lowest overall amount of parent-reported screen time compared to children in families with an indulgent or neglectful parenting style, our results indicated that the magnitude of the association between parenting style and children's screen time is relatively modest. Additionally, we investigated whether parenting style within the household might be an effect-modifier in the association between the social and physical home environment and screen time of the children. Parenting style within the household also appeared not to be an effect-modifier in any of the associations between the social or physical home environment characteristics and screen time (p-values >0.10 for all interaction terms, data not shown). This indicates independent associations between the social and physical home environment and children's screen time. A study among older children (aged 10–11 years), however, reported that permissive parenting (comparable with an indulgent parenting style) was associated with a higher level of watching TV compared to authoritative parenting [17]. Further, studies on energy intake among 6 to 8 year-olds [27] and 12 to 17 year-olds [35] also reported more pronounced effects of parenting practices on children's energy intake among households with an authoritative parenting style. Therefore, more longitudinal studies are needed to investigate a potential long-term effect of parenting style on children's screen time.

In 2007–2008, 8.7% of the 5-year-olds in our study population had a TV in their bedroom and 15.3% had a computer or game console in their bedroom. It is likely that nowadays these percentages are higher. In the present study, a TV and computer or game console in the bedroom was associated with a higher odds ratio for watching TV more than 2 hours a day and spending more than 30 minutes per day on computers or game consoles. In a qualitative study investigating the thought-process of parents behind having a TV in the child's bedroom [46], it was reported that parents think that it assists with bedtime routine (i.e. children are in their bedroom and can watch TV until it is time for them to go to sleep), that it allows family members to each watch what they want, and that it stops fighting amongst children. It might be useful for interventions to discuss these incorrect notions of parents. Further, the study also indicated that once a TV is present in a child's bedroom it is difficult to remove and, therefore, it might be better to prevent the placement of a TV in the child's bedroom in the first place.

Our study provides new insights into the associations between parenting style, the home environment and children's screen time. The social and physical home environment has unique effects on children's screen time that are independent of parenting style. Our results indicate a relative modest association between parenting style and screen time at the age of 5 years. To reduce the time a child spends watching TV or using a computer or game console, it might be important to make parents more aware of the influence they have on their child's behavior, especially when the child is young. However, parents might find it an increasing challenge to limit their children's screen time because the changes in society increasingly promote children's screen time [47], [48]; for example the availability of multiple TV channels around the clock with programs for children, the increase in computer games aimed at children, but also the increase in use of electronic media in children's education. For these reasons, parents might experience it as a challenge to create a home environment that limits screen time. Therefore, it might be important that interventions aiming to reduce children's screen time address the social and physical environmental context in which children's screen time occurs. Such interventions might be most effective if they start during early childhood and before family habits are established. These interventions should improve the ability of parents to create and maintain a healthy home environment by providing the parents with information, skills, support, and encouragement to make changes in parenting practices and in the physical home environment. Future studies are needed to evaluate whether interventions that focus on improving the social and physical home environment (e.g. by promoting the introduction of family rules or ‘passive controls’ regarding screen time – for example software programs that restrict access to the TV or computer or game console, by preventing the placement of a TV or computer or game console in (young) children's bedrooms, but also by suggesting alternative activities such as drawing or playing outside) indeed result in a reduction of the children's screen time.

## Supporting Information

Appendix S1 **Items assessing parenting style and the social and physical home environment.**
(DOC)

Appendix S2 **Logistic regression analyses for the association between the parenting style dimension involvement and using computers or game consoles >30 min/day by the child, and the association after adjustment for social and physical environment characteristics (n = 3067).**
(DOC)
