# Supplementary material for: Parenting Style, the Home Environment, and Screen Time of 5-Year-Old Children; The ‘Be Active, Eat Right’ Study
Source: PLoS One. 2014 Feb 12;9(2):e88486. doi: 10.1371/journal.pone.0088486 (PMC3922818; doi:10.1371/journal.pone.0088486)
Supplement: Appendix S1 — Items assessing parenting style and the social and physical home environment. (DOC) [file pone.0088486.s001.doc]

Appendix 1 Items assessing parenting style and the social and physical home environment

| **Scale** | Scale properties | Scale description | Translated items | Item response scale |
| --- | --- | --- | --- | --- |
| Parenting style | | | | |
| Dimension involvement | Cronbach’s alpha 0.75 | General parenting style dimension involvement | - When someone within our family comes home or leaves home, than he or she let other family members know | 1 =strongly agree to 5 = strongly disagree |
|  | Scale range 1-5 |  | - I encourage my child to try harder when he or she receives a poor grade in school | 1 = strongly agree to 5 = strongly disagree |
|  |  |  | - I help my child with an assignment that he or she does not understand | 1 = strongly agree to 5 = strongly disagree |
|  |  |  | - My child can count on me when he or she has some kind of problem | 1 = strongly agree to 5 = strongly disagree |
|  |  |  | - I find it very easy to talk openly with my child | 1 = strongly agree to 5 = strongly disagree |
|  |  |  | - I spend time just talking with my child | 1 = strongly agree to 5 = strongly disagree |
|  |  |  | - When my child receives a good grade in school, I show him or her my approval | 1 = strongly agree to 5 = strongly disagree |
|  |  |  | - We do things for fun together regularly as a family | 1 = strongly agree to 5 = strongly disagree |
|  |  |  | - When my child gets a poor grade in school, I suggest to help | 1 = strongly agree to 5 = strongly disagree |
| Dimension strictness | Cronbach’s alpha 0.78 | General parenting style dimension strictness | - I really know what my child does in her or his free time | 1 = strongly agree to 5 = strongly disagree |
|  | Scale range 1-5 |  | - I try to know where my child is in the afternoon after school | 1 = strongly agree to 5 = strongly disagree |
|  |  |  | - I really know where my child goes at night | 1 = strongly agree to 5 = strongly disagree |
|  |  |  | - I really know where my child is in the afternoon after school | 1 = strongly agree to 5 = strongly disagree |
|  |  |  | - I try to know where my child goes at night | 1 = strongly agree to 5 = strongly disagree |
|  |  |  | - I try to know what my child does in her or his free time | 1 = strongly agree to 5 = strongly disagree |
| Social home environment (parenting practices) | | | | |
| Family rules about watching TV | Index range 1-3 | Number of rules about watching TV by the child reported by the parents | Do you have rules in your household about: |  |
|  |  |  | - When your child is allowed to watch TV? | no/yes |
|  |  |  | - How long your child is allowed to watch TV? | no/yes |
| Family rules about using computers or game consoles | Index range 1-3 | Number of rules about using computers or game consoles by the child reported by the parents | Do you have rules in your household about: |  |
|  |  |  | - When your child is allowed to use a computer or game console? | no/yes |
|  |  |  | - How long your child is allowed to use a computer or game console? | no/yes |
| Parental monitoring watching TV | - | Parental monitoring concerning watching TV by the child | To what extent do you monitor for how long your child watches TV? | 1 = never to 5 = always |
| Parental monitoring using computers or game consoles | - | Parental monitoring concerning using computers or game consoles by the child | To what extent do you monitor for how long your child uses computers or game consoles? | 1 = never to 5 = always |
| Parental urging TV | - | Parents urge their child to turn off the TV | How often do you tell your child to turn off the TV? | 1 = never to 5 = always |
| Parental urging computer or game console | - | Parents urge their child to turn off the computer or game console | How often do you tell your child to turn off the computer or game console? | 1 = never to 5 = always |
| Child autonomy watching TV | Cronbach’s alpha 0.90  Scale range 1-5 |  | How often is your child allowed to decide for himself or herself: |  |
|  |  |  | - Whether he or she watches TV? | 1 = never to 5 = always |
|  |  |  | - When he or she watches TV? | 1 = never to 5 = always |
|  |  |  | - For how long he or she watches TV? | 1 = never to 5 = always |
| Child autonomy using computers or game consoles | Cronbach’s alpha 0.94  Scale range 1-5 |  | How often is your child allowed to decide for himself or herself: |  |
|  |  |  | - Whether he or she uses a computer or game console? | 1 = never to 5 = always |
|  |  |  | - When he or she uses a computer or game console? | 1 = never to 5 = always |
|  |  |  | - For how long he or she uses a computer or game console? | 1 = never to 5 = always |
| Physical home environment | | | | |
| Nr. of TVs in household | Scale range 1-5 | Number of TVs that are present in the household | How many TVs do you have in your household? | 1 = 0 TVs to 5 = 4 TVs or more |
| TV in child’s bedroom | - | Availability of a TV in the child’s bedroom | Does your child have a TV in his or her bedroom? | no/yes |
| Nr. of computers or game consoles in the household | Scale range 1-5 | Number of computers or game consoles in the household | How many computers or game consoles do you have in your household? | 1 = 0 computers or game consoles to 5 = 4 computers or game consoles or more |
| Computer or game console in child’s bedroom | - | Availability of a computer or game console in the child’s bedroom | Does your child have a computer or game console in his or her bedroom? | no/yes |
