# Supplementary material for: Parenting Style, the Home Environment, and Screen Time of 5-Year-Old Children; The ‘Be Active, Eat Right’ Study
Source: PLoS One. 2014 Feb 12;9(2):e88486. doi: 10.1371/journal.pone.0088486 (PMC3922818; doi:10.1371/journal.pone.0088486)
Supplement: Appendix S2 — Logistic regression analyses for the association between the parenting style dimension involvement and using computers or game consoles >30 min/day by the child, and the association after adjustment for social and physical environment characteristics (n = 3067). (DOC) [file pone.0088486.s002.doc]

Appendix 2 Logistic regression analyses for the association between the parenting style dimension involvement and using computers or game consoles >30 min/day by the child, and the association after adjustment for social and physical environment characteristics (n = 3067)

|  | Using computers or game consoles >30 min/day,  OR (95% CI) | Change^a^ |
| --- | --- | --- |
| Parenting style dimension involvement (basic model) | 1.34 (1.02 – 1.77) |  |
| Basic model + social home environment (parenting practices) |  |  |
| Nr. of family rules about using computers or game consoles | 1.31 (1.00 – 1.74) | -8.8% |
| Parental monitoring concerning using computers or game consoles | 1.29 (0.98 – 1.71) | -14.7% |
| Parental urging to turn off computer or game console | 1.26 (0.95 – 1.66) | -23.5% |
| Child autonomy concerning using computers or game consoles | 1.41 (1.07 – 1.86) | +20.6% |
| Basic model + physical home environment |  |  |
| Nr. of computers or game consoles in household | 1.33 (1.00 – 1.77) | -2.9% |
| Child has computer or game console in bedroom | 1.32 (1.00 – 1.74) | -5.9% |

For details on the measures used, see Appendix 1.

The ORs are the pooled results of analysis of the five imputed datasets.

The ORs are adjusted for sociodemographic characteristics (sex and age of the child, ethnic background of the child, educational level of the parent, employment status and family structure).

^a^ Change represent the change in OR relative to the basic model after adjustment for the characteristics of the social and physical home environment ([OR_basic model+characteristic_ – OR_basic model_]/[OR_basic model_ – 1] x 100) .
